# Treatment of Extended Kalman Filter Implementations for the Gyroless Star Tracker

**DOI:** 10.3390/s22229002

**Published:** 2022-11-21

**Authors:** Joshua J. R. Critchley-Marrows, Xiaofeng Wu, Iver H. Cairns

**Affiliations:** 1 School of Aerospace, Mechanical and Mechatronic Engineering, The University of Sydney, Sydney, NSW 2040, Australia; 2 School of Physics, The University of Sydney, Sydney, NSW 2040, Australia

**Keywords:** attitude, star tracker, Kalman Filter, Static Attitude Estimation, SSA

## Abstract

The literature since Apollo contains exhaustive material on attitude filtering, usually treating the problem of two sensors, a combination of state measuring and inertial devices. More recently, it has become popular for a sole attitude determination device to be considered. This is especially the case for a star tracker given its unbiased stellar measurement and recent improvements in optical sensor performance. The state device indirectly estimates the attitude rate using a known dynamic model. In estimation theory, two main attitude filtering approaches are classified, the additive and the multiplicative. Each refers to the nature of the quaternion update in the filter. In this article, these two techniques are implemented for the case of a sole star tracker, using simulated and real night sky image data. Both sets of results are presented and compared with each other, with a baseline established through a basic linear least square estimate. The state approach is more accurate and precise for measuring angular velocity than using the error-based filter. However, no discernible difference is observed between each technique for determining pointing. These results are important not only for sole device attitude determination systems, but also for space situational awareness object localisation, where attitude and rate estimate accuracy are highly important.

## 1. Introduction

To improve attitude determination performance, statistical estimation and filtering techniques are adopted by the space system designer. The most prevalent filter is the Kalman Filter, proposed by Rudolf Kalman during the 1950s [1]. The theory was developed and applied to space flight through a series of NASA reports [2,3] during the early 1960s. The earliest known study to apply the Kalman Filter for the attitude determination problem was in 1970 [4], where Farrell was one of the first to recognise the merit of the technique.

Since the Apollo era, the Kalman Filter and its modifications have been proposed for a variety of attitude determination systems, each adopting a different set of attitude determination sensors. The work by Farrell studied the crude use of sun sensors and magnetometers [4]. One of the first uses of a star sensor was used with a gyroscope by the Aerospace Corporation [5], applying a discrete Kalman Filter.

During the space race, satellite applications in low and medium Earth orbits were discovered. Since many missions required only an Earth-pointing attitude, not experiencing many complex dynamic motion routines, the gyroscope was neglected, and the attitude calculated using orientation only sensors. Gai in the 1980s [6] attempted to use a star sensor that, at the time, could only receive a single star measurement at a fast enough rate for attitude estimation. The attitude accuracy was poor, applying a simple finite difference-based approach and assuming negligible noise.

Spacecraft torques in the control system loop, as well as external torques caused by the space environment such as solar wind or aerodynamic drag [7,8], have also been considered in the estimation problem. However, it was recognised the algorithms were still not robust enough to model errors or remove the limitations raised by Gai [6] for angular velocity estimation by finite difference. Modelling external torques is considered outside the scope of this article, requiring position knowledge. They are also typically considered in combined systems with an environmental sensor, such as a magnetometer [9,10].

In recent decades, attempts at formalising a standard Extended Kalman Filter approach for spacecraft attitude determination were made by Crassidis, Markley, Shuster, et al. [9,11,12,13,14]. Attitude is typically expressed by the quaternion, which avoids issues in singularities and sequential rotation ordering common for Euler angle and matrix representations. However, quaternions must maintain certain associative properties as well as a unitary norm, which can be difficult to assure in estimation and filtering problems. These approaches utilise multiple sensors.

Approaches to the filtration problem are distinguished between additive and multiplicative operations on the attitude quaternion. A section of *Fundamentals of Spacecraft Attitude Determination and Control* was compiled by these authors, where different approaches were considered [15]. The methodology implements a magnetometer or star tracker for orientation estimation, alongside a gyroscope for rate estimation.

Contemporary literature has revisited this problem given recent performance improvements in optical and processor technology [16,17,18,19,20]. These systems permit tracking of multiple stellar sources at a high rate and resolution, permitting increased precision, accuracy and update rates of the attitude estimate. These improvements have allowed for the star tracker to be used as the sole instrument for attitude determination, a gyroless star tracker, permitting for unbiased attitude and rate estimates. It is also more compact and power efficient, ideal parameters for optimisation by the satellite designer. Early work by [21] was an early attempt of a gyroless star tracker Extended Kalman Filter (EKF) utilising multiple star measurements, but used a more primitive EKF implementation that did not consider the advanced formalisation in [15].

Gyroless star trackers are becoming increasingly important for Space Situational Awareness (SSA) applications [22,23]. Dual-use star trackers are in development to support SSA. In their operation, the attitude estimate of the star tracker is important to determine the observed space object’s orbit. The conclusions of this work are thus important to this emerging field of star tracker application, where the relevance and importance of rate estimation moves beyond just attitude control requirements.

Recent advancements considering a gyroless star tracker in the Kalman filter, acting as the only sensor, are more limited. This is especially the case with implementations that treat all star measurements within the filter measurement vector. [24] considers a multiplicative EKF alongside a Singular Value Decomposition (SVD) state estimate for a gyroless star tracker, but utilising control elements for rate estimation. This approach does not utilise all star measurements in the filter, but the SVD derived attitude estimate. Similar work by [25] also contain similar constraints.

The gyroless star tracker is considered in an additive-based Kalman filter by [17,18]. A disadvantage of the additive approach is that it does not maintain unity of the quaternion norm. Modern approaches re-normalise the quaternion after each update to assure unity is maintained. This has been criticised since it always creates some error in the quaternion estimate, limiting performance. However, it can be argued that the error is negligible compared to other error sources from the camera and attitude propagation.

Filter design, including the Kalman filter for guidance systems, has advanced significant recently with the adoption of machine learning [26,27,28]. However, there is great concern that machine learning cannot assure the integrity required for satellite attitude determination, where neural networks act like a ‘black box’. They might be considered for Earth observation, rover guidance and mission planning [29].

This work treats angular velocity as constant, and makes the assumption that any angular acceleration is negligible. Generally, a non-negligible angular acceleration would only take place if a control torque is acted on the satellite. Thus, the expected angular acceleration is already known by the system.

The Calculated Reference of Stellar System (CROSS) star tracker is used as a test platform in this article. CROSS is a Wide Field of View (WFOV) star tracker being developed by the University of Sydney that seeks to be a high performing, versatile and competitive attitude determination unit for spacecraft designers [30,31,32].

In this paper, the additive and multiplicative EKF are applied to evaluate and determine the best approach to the gyroless star tracker attitude estimation problem. The novelty and originality of the work are summarised by:

1. Novel implementation of the multiplicative EKF for the case of the gyroless star tracker.
2. An in-depth, independent analysis to the state-of-the-art additive EKF for the gyroless star tracker by [17,18], comparing to the multiplicative EKF approach as well as an unfiltered approach (i.e., Linear Least Squares (LLS) only).
3. Analysis employs both simulation and real night sky testing. Provides a case example of the novel approach of star tracker testing by [33].

The significance of the research is summarised as:

- Enabling more compact and lower power attitude determination systems.
- Improving attitude estimation accuracy and precision, a topic of interest for not only satellite attitude control but SSA applications.
- Reducing bias to attitude and rate estimates.

The paper is structured by firstly outlining both EKF models. It then implements each model by simulation and real night-sky testing, as well as comparing to a static LLS approach, to discuss and evaluate the best approach. Theoretical arguments are also considered in this discussion. Recommendations of the best approach are made at the conclusion.

Given that gyroscope bias is now removed, and the EKF is estimating the angular velocity state, additive and multiplicative filters are relabelled as the state and the state-error EKF. This naming change highlights that the multiplicative approach estimates the state error, rather than directly the state.

## 2. Model and Methods

This section introduces the conventions adopted to describe the spacecraft attitude. A sensor model is then introduced for the star tracker, which includes star position error considerations. The section closes by describing the two approaches to the EKF implementation, which contains a modified version of implementations in [15,17] to treat the performance of the gyroless star tracker case.

### 2.1. Attitude Definitions

Attitude is typically expressed as a matrix that transforms a vector from one coordinate frame to another. In the case of spacecraft attitude, the transformation typically relates the sensor or spacecraft frame to a celestial body frame, such as for the Earth or Solar System centre. The celestial frame will be denoted by r, and the sensor or spacecraft frame will be denoted by b. The relation between each frame and the attitude may be written as,

(1) b=Ar,

where *A* is the attitude matrix.

**Figure 1:**
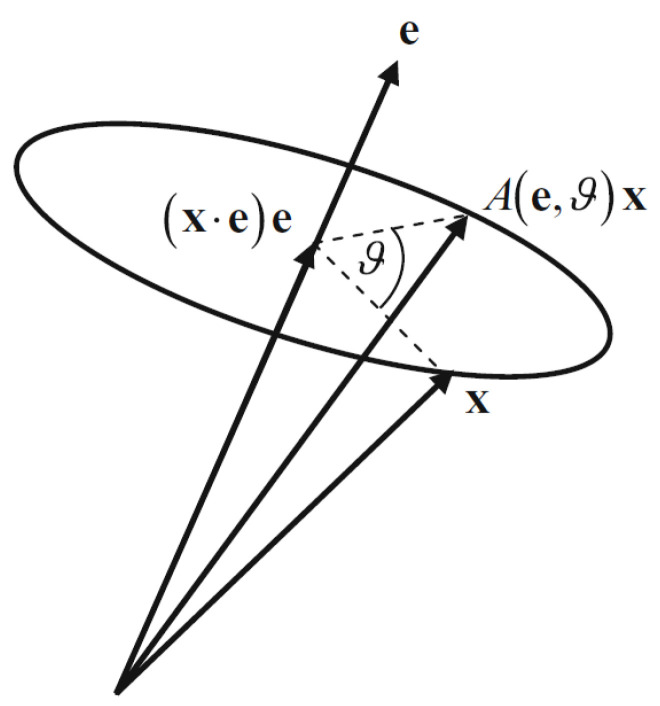
Rotation of an arbitrary vector x about the rotation vector [15].

To aid in the representation of attitude, consider an axis e for a rotation to act on. The angle of rotation may then be denoted by θ. The rotation of an arbitrary vector x about the rotation vector is illustrated in Figure 1. The attitude matrix may be expressed as a function of the rotation vector and angle, A=A(e,θ).

It is more convenient, however, to express the rotation as a quaternion. The quaternion q may be expressed in terms of the rotation vector as,

(2) $$q=\left\{\begin{array}{c} q_{0}\\ q_{1}\\ q_{2}\\ q_{3} \end{array}\right\}=\left\{\begin{array}{c} \cos\frac{\theta }{2}\\ e\sin\frac{\theta }{2} \end{array}\right\}.$$

The quaternion must satisfy the unity constraint |q|=1. Maintaining this constraint in estimation and filtering can prove problematic, as introduced in Section 1.

The attitude matrix and quaternion are related by,

(3) $$A=\left[\begin{array}{ccc} q_{0}^{2}+q_{1}^{2}-q_{2}^{2}-q_{3}^{2} & 2(q_{1}q_{2}-q_{0}q_{3}) & 2(q_{1}q_{3}+q_{0}q_{2})\\ 2(q_{1}q_{2}+q_{0}q_{3}) & q_{0}^{2}-q_{1}^{2}+q_{2}^{2}-q_{3}^{2} & 2(q_{2}q_{3}-q_{0}q_{1})\\ 2(q_{1}q_{3}-q_{0}q_{2}) & 2(q_{2}q_{3}+q_{0}q_{1}) & q_{0}^{2}-q_{1}^{2}-q_{2}^{2}+q_{3}^{2} \end{array}\right].$$

Derivations of attitude kinematics are not included in this description, as they are extensively written in the literature [15,34], but are instead stated. The time derivative of the attitude quaternion may be expressed in terms of the angular velocity, $\omega ={\left\{\begin{array}{ccc} \omega_{x} & \omega_{y} & \omega_{z} \end{array}\right\}}^{T}$, by,

(4) $$\dot{q}\left(t\right)=\frac{1}{2}\omega \left(t\right)⊗q\left(t\right)=\frac{1}{2}\Omega \left(\omega \left(t\right)\right)q\left(t\right),$$

where Ω is a matrix representation of the tensor product operation between the angular velocity and quaternion,

(5) $$\Omega \left(\omega \right)=\left[\begin{array}{cccc} 0 & -\omega_{x} & -\omega_{y} & -\omega_{z}\\ \omega_{x} & 0 & -\omega_{z} & \omega_{y}\\ \omega_{y} & \omega_{z} & 0 & -\omega_{x}\\ \omega_{z} & -\omega_{y} & \omega_{x} & 0 \end{array}\right].$$

A linearised form of the state transition over the duration of a time step Δt can be derived. Assuming a constant angular velocity over the time step and that the angular rotation is small, the closed form may be expressed as,

(6) $$q\left(t+\Delta t\right)=\left[\cos\left(\frac{|\omega (t)|\Delta t}{2}\right)I_{4}+\frac{1}{\left|\omega (t)\right|}\sin\left(\frac{|\omega (t)|\Delta t}{2}\right)\Omega \right]q\left(t\right)=\Phi_{qq}q\left(t\right),$$

where $\Phi_{qq}$ is the quaternion transition matrix and $I_{4}$ is the 4×4 identity matrix.

The angular velocity may also be estimated assuming it is constant over the time step. The quaternion rate is expressed as,

(7) $$\dot{q}=\lim_{\Delta t\to 0}\frac{q(t+\Delta t)-q(t)}{\Delta t}.$$

The quaternion rate may then be estimated using this equation if the time step is justifiably small. Equation (4) may then be rearranged in terms of the angular velocity, using a similar approach to Equation (6),

(8) $$\omega \left(t\right)=2\Xi^{T}\left(q\left(t\right)\right)\dot{q},$$

where,

(9) $$\Xi \left(q\right)=\left[\begin{array}{ccc} -q_{1} & -q_{2} & -q_{3}\\ q_{0} & -q_{3} & q_{2}\\ q_{3} & q_{0} & -q_{1}\\ -q_{2} & q_{1} & q_{0} \end{array}\right].$$

### 2.2. Star Tracker Model

The star tracker captures images of the stars using a standard camera and lens assembly. The image is then analysed to identify stars against a known catalogue. Using the measured unit vectors of stars in a sensor frame and the known unit vectors from a catalogue in a global frame, an attitude may be calculated.

The measured star image is represented by the sub-pixel coordinates of the stellar source (α,γ). The sensor frame unit vector is related to the sub-pixel coordinates by,

(10) $$\alpha =-f\frac{b_{x}}{b_{z}},\;\gamma =-f\frac{b_{y}}{b_{z}},$$

where *f* is the focal length. The body and celestial frame unit vectors are then related by Equation (1).

The reverse relation to Equation (10) uses the unit vector normalisation condition, |b|=1. So, the body unit vector may be calculated by,

(11) $$b=\frac{1}{\sqrt{\alpha^{2}+\gamma^{2}+f^{2}}}\left\{\begin{array}{c} -\alpha \\ -\gamma \\ f \end{array}\right\}.$$

A common measurement error model for the measured image coordinates α and γ is [11,14],

(12) $$R=\frac{\sigma^{2}}{1+\alpha^{2}+\beta^{2}}\left[\begin{array}{cc} {\left(1+\alpha^{2}\right)}^{2} & {\left(\alpha \gamma \right)}^{2}\\ {\left(\alpha \gamma \right)}^{2} & {\left(1+\gamma^{2}\right)}^{2} \end{array}\right].$$

This model is appropriate for sensor measurements that are no greater than $15^{\circ }$ from the boresight. The star tracker measurements considered in this paper are $10^{\circ }$ from boresight. The eigenvalues and eigenvectors can be determined from the matrix to know the maximum error of the image.

### 2.3. Extended Kalman Filter Model

Two approaches to applying EKF to attitude determination systems are considered in the literature, the state-based and state error-based, as are first introduced in Section 1.

A stand-alone star tracker will be initially introduced, using the state-based approach. The popular error state variant will then be discussed. The angular velocity cannot be directly measured by the star tracker. An estimate by finite difference, applying Equation (8), is adopted. The EKF is then compared to static estimation by LLS, using the popular Davenport q method described in [15].

#### 2.3.1. State-Based Estimation

Using a stand-alone star tracker in the attitude determination system may estimate both the attitude as a quaternion and the angular velocity. This section adopts approaches initially proposed in [17].

The state variables may be expressed as,

(13) $$x={\left\{\begin{array}{cc} q^{T} & \omega^{T} \end{array}\right\}}^{T},$$

where $q={\left[\begin{array}{cccc} q_{0} & q_{1} & q_{2} & q_{3} \end{array}\right]}^{T}$ and $\omega ={\left[\begin{array}{ccc} \omega_{x} & \omega_{y} & \omega_{z} \end{array}\right]}^{T}$. The state transition, or propagation update, may be modelled using,

(14) x(t+Δt)=ϕ(x(t))+σ(t),

where ϕ is the state transition function and σ is the additive Gaussian noise. Using Equation (6), the state transition function may be expressed as,

(15) $$\phi \left(x\left(t\right)\right)=\left\{\begin{array}{c} \Phi_{qq}q\left(t\right)\\ \omega \end{array}\right\}$$

The EKF is an approach to using non-linear system models in the linear Kalman Filter technique. It does this by a process of linearisation, as has already been applied to Equation (6). The overall state transition matrix is represented by,

(16) $$\Phi =\left[\begin{array}{cc} \frac{\partial q(t+\Delta t)}{\partial q(t)} & \frac{\partial q(t+\Delta t)}{\partial \omega (t)}\\ \frac{\partial \omega (t+\Delta t)}{\partial q(t)} & \frac{\partial \omega (t+\Delta t)}{\partial \omega (t)} \end{array}\right]=\left[\begin{array}{cc} \Phi_{qq} & \Phi_{q\omega }\\ \Phi_{\omega q} & \Phi_{\omega \omega } \end{array}\right],$$

where $\Phi_{qq}$ is already known from Equation (6). The partial derivative of q(t+Δt) with respect to ω(t) is,

(17) $$\Phi_{q\omega }=\frac{\partial q(t+\Delta t)}{\partial \omega (t)}=\frac{\partial \Phi_{qq}q\left(t\right)}{\partial \omega (t)},$$

and thus for i=x,y,z,

(18) $$\frac{\partial \Phi_{qq}q\left(t\right)}{\partial \omega (t)}=\left[\frac{-\omega_{i}\Delta t}{2|\omega |}\sin\left(\frac{|\omega |\Delta t}{2}\right)I_{4}+\left(\frac{\omega_{i}\Delta t}{{2|\omega |}^{2}}\cos\left(\frac{|\omega |\Delta t}{2}\right)-\frac{\omega_{i}}{{\left|\omega \right|}^{3}}\sin\left(\frac{|\omega |\Delta t}{2}\right)\right)\Omega +\frac{1}{\left|\omega \right|}\sin\left(\frac{|\omega |\Delta t}{2}\right)\frac{\partial \Omega }{\partial \omega_{i}}\right]q\left(t\right),$$

where,

$$\frac{\partial \Omega }{\partial \omega_{x}}=\left[\begin{array}{cccc} 0 & -1 & 0 & 0\\ 1 & 0 & 0 & 0\\ 0 & 0 & 0 & -1\\ 0 & 0 & 1 & 0 \end{array}\right],\;\frac{\partial \Omega }{\partial \omega_{y}}=\left[\begin{array}{cccc} 0 & 0 & -1 & 0\\ 0 & 0 & 0 & 1\\ 1 & 0 & 0 & 0\\ 0 & -1 & 0 & 0 \end{array}\right],\;\frac{\partial \Omega }{\partial \omega_{z}}=\left[\begin{array}{cccc} 0 & 0 & 0 & -1\\ 0 & 0 & -1 & 0\\ 0 & 1 & 0 & 0\\ 1 & 0 & 0 & 0 \end{array}\right].$$

Since ω is assumed to be constant over the time step, $\Phi_{\omega q}=0$ and $\Phi_{\omega \omega }=I_{3}$.

In a similar form to the state or propagation update, as in Equation (14), the measurement update is given by,

(19) $$\hat{b}=h\left(x\right)+v,$$

where h is the measurement function and v is the measurement noise, modelled as a zero-mean Gaussian noise. The measurements of the star tracker are given in sub-pixel coordinates calculated from the star source brightness distribution.

The expected measurement h(x) is derived from the identified stars. The star catalogue coordinates are given in the inertial frame r. These are transformed to the star tracker sensor frame by the attitude matrix *A* using Equation (1). The ith star locations known in the derived expected image with coordinates $\alpha_{i}$ and $\gamma_{i}$ are obtained using Equation (10).

A process of linearisation is also required for the measurement update, and so the partial derivative of the measurement function with respect to the state variable produces the measurement matrix,

(20) $$H\left(x\right)=\frac{\partial h(x)}{\partial x}=\left[\begin{array}{ccccccc} \frac{\partial \alpha_{1}}{\partial q_{0}} & \frac{\partial \alpha_{1}}{\partial q_{1}} & \frac{\partial \alpha_{1}}{\partial q_{2}} & \frac{\partial \alpha_{1}}{\partial q_{3}} & \frac{\partial \alpha_{1}}{\partial \omega_{x}} & \frac{\partial \alpha_{1}}{\partial \omega_{y}} & \frac{\partial \alpha_{1}}{\partial \omega_{z}}\\ \vdots & \vdots & \vdots & \vdots & \vdots & \vdots & \vdots \\ \frac{\partial \gamma_{n}}{\partial q_{0}} & \frac{\partial \gamma_{n}}{\partial q_{1}} & \frac{\partial \gamma_{n}}{\partial q_{2}} & \frac{\partial \gamma_{n}}{\partial q_{3}} & \frac{\partial \gamma_{n}}{\partial \omega_{x}} & \frac{\partial \gamma_{n}}{\partial \omega_{y}} & \frac{\partial \gamma_{n}}{\partial \omega_{z}} \end{array}\right],$$

where *n* is the total number of stars in the field of view. Using the chain rule, the partial derivatives with each state variable may be calculated using,

(21) $$\begin{array}{cc} \frac{\partial \alpha }{\partial x} & =\frac{\partial \alpha }{\partial b_{x}}\frac{\partial b_{x}}{\partial x}+\frac{\partial \alpha }{\partial b_{y}}\frac{\partial b_{y}}{\partial x}+\frac{\partial \alpha }{\partial b_{z}}\frac{\partial b_{z}}{\partial x}, \end{array}$$

(22) $$\begin{array}{cc} \frac{\partial \gamma }{\partial x} & =\frac{\partial \gamma }{\partial b_{x}}\frac{\partial b_{x}}{\partial x}+\frac{\partial \gamma }{\partial b_{y}}\frac{\partial b_{y}}{\partial x}+\frac{\partial \gamma }{\partial b_{z}}\frac{\partial b_{z}}{\partial x}. \end{array}$$

So, the partial derivative of the measured sub-pixel star locations α and β are,

$$\frac{\partial \alpha }{\partial b_{x}}=-\frac{f}{b_{z}},\;\frac{\partial \alpha }{\partial b_{y}}=0,\;\frac{\partial \alpha }{\partial b_{z}}=\frac{b_{x}f}{b_{z}^{2}},\;\frac{\partial \gamma }{\partial b_{x}}=0,\;\frac{\partial \gamma }{\partial b_{y}}=-\frac{f}{b_{z}},\;\frac{\partial \gamma }{\partial b_{z}}=\frac{b_{y}f}{b_{z}^{2}}.$$

The partial derivative of b with respect to the angular velocity ω is,

(23) $$\frac{\partial b}{\partial \omega }=0,$$

and with respect to the quaternion q is,

(24) $$\frac{\partial b}{\partial q}=\frac{\partial A}{\partial q}r,$$

where for each quaternion the partial derivative of the attitude matrix is,

$$\begin{array}{c} \frac{\partial A}{\partial q_{0}}=\left[\begin{array}{ccc} 2q_{0} & 2q_{3} & -2q_{2}\\ -2q_{3} & 2q_{0} & 2q_{1}\\ 2q_{2} & -2q_{1} & 2q_{0} \end{array}\right],\;\frac{\partial A}{\partial q_{1}}=\left[\begin{array}{ccc} 2q_{1} & 2q_{2} & 2q_{3}\\ 2q_{2} & -2q_{1} & 2q_{0}\\ 2q_{3} & -2q_{0} & -2q_{1} \end{array}\right],\\ \frac{\partial A}{\partial q_{2}}=\left[\begin{array}{ccc} -2q_{2} & 2q_{1} & -2q_{0}\\ 2q_{1} & 2q_{2} & 2q_{3}\\ 2q_{0} & 2q_{3} & -2q_{2} \end{array}\right],\;\frac{\partial A}{\partial q_{3}}=\left[\begin{array}{ccc} -2q_{3} & 2q_{0} & 2q_{1}\\ -2q_{0} & -2q_{3} & 2q_{2}\\ 2q_{1} & 2q_{2} & 2q_{3} \end{array}\right]. \end{array}$$

The complete methodology of the EKF model is summarised by Table 1. The + and − superscripts indicate the pre- and post-measurement update, respectively, used by the state vector x and covariance matrix *P*. The precidicted state vector is expressed by z.

Given the risk to ill-conditioning of the covariance matrix *P* owing to non-linearities and the re-normalisation of the quaternion, *P* is checked for positive definiteness by a Cholesky Factorisation attempt. If the attempt failed, the covariance matrix is reset by negating all diagonal negative terms and setting all non-diagonal terms to zero. In the reported results of this work, this reset was never necessary.

#### 2.3.2. State Error Estimation

For the stand-alone star tracker, the state error estimation approach uses the formalisation from [15]. However, the approach is made novel by replacing the gyroscope bias state by a direct estimate of the angular velocity, making various modifications to the model to reflect this state estimate change. The state variables may be expressed as in the state-based approach,

(25) $$x={\left\{\begin{array}{cc} q^{T} & \omega^{T} \end{array}\right\}}^{T}.$$

However, the filter will not act directly on the state, but the state errors. The state error vector is expressed as,

(26) $$\Delta x={\left\{\begin{array}{cc} \delta \theta^{T} & \delta \omega^{T} \end{array}\right\}}^{T},$$

where $\delta \theta ={\left\{\begin{array}{ccc} \delta \theta_{x} & \delta \theta_{y} & \delta \theta_{z} \end{array}\right\}}^{T}$ and $\delta \omega ={\left\{\begin{array}{ccc} \delta \omega_{x} & \delta \omega_{y} & \delta \omega_{z} \end{array}\right\}}^{T}$.

The propagation update is rewritten as,

(27) Δx(t+Δt)=ϕ(Δx(t))+σ(t).

The linearised state transition matrix is then,

(28) $$\Phi =\left[\begin{array}{cc} \frac{\partial \delta \theta (t+\Delta t)}{\partial \delta \theta (t)} & \frac{\partial \delta \theta (t+\Delta t)}{\partial \delta \omega (t)}\\ \frac{\partial \delta \omega (t+\Delta t)}{\partial \delta \theta (t)} & \frac{\partial \delta \omega (t+\Delta t)}{\partial \delta \omega (t)} \end{array}\right]=\left[\begin{array}{cc} \Phi_{\delta \theta \delta \theta } & \Phi_{\delta \theta \delta \omega }\\ \Phi_{\delta \omega \delta \theta } & \Phi_{\delta \omega \delta \omega } \end{array}\right].$$

An alternative approach is adopted by [15] to derive the state transition matrix. Using the linearised state space estimate equation for the rate of state change,

(29) $$\Delta \dot{\hat{x}}\left(t\right)=F\left(t\right)\hat{x}\left(t\right),$$

where F(t) is the Fisher information matrix, which provides a linearised matrix operator on the propagated state via,

(30) $$F\left(t\right)=\frac{\partial \dot{\hat{x}}\left(t\right)}{\partial \hat{x}\left(t\right)}.$$

It is known from [15] that the rate of attitude error change is related to the attitude error and angular velocity terms by,

(31) $$\delta \dot{\theta }=-\left[\hat{\omega }\times \right]\delta \theta +\delta \omega ,$$

where $\left[\hat{\omega }\times \right]$ is the matrix operator of the cross product, written as,

(32) $$\left[\hat{\omega }\times \right]=\left[\begin{array}{ccc} 0 & \hat{\omega_{z}} & -\hat{\omega_{y}}\\ -\hat{\omega_{z}} & 0 & \hat{\omega_{x}}\\ \hat{\omega_{y}} & -\hat{\omega_{x}} & 0 \end{array}\right].$$

So the Fisher information matrix is expressed as,

(33) $$F\left(t\right)=\left[\begin{array}{cc} -[\hat{\omega }\times ] & I_{3}\\ 0_{3} & 0_{3} \end{array}\right],$$

where $I_{3}$ is the 3×3 identity matrix. The Fisher information matrix and the transition matrix is related by the expression,

(34) $$\frac{d}{dt}\Phi =F\left(t\right)\Phi .$$

Considering each side and maintaining equivalence, the transition matrix constituents may be expressed as,

$$\begin{array}{cc} \; & \Phi_{\delta \theta \delta \theta }=I_{3}-\left[\hat{\omega }\times \right]\frac{\sin(|\hat{\omega }|\Delta t)}{|\hat{\omega }|}+{\left[\hat{\omega }\times \right]}^{2}\frac{\left[1-\cos(|\hat{\omega }|\Delta t)\right]}{|\hat{\omega }{|}^{2}},\\ \; & \Phi_{\delta \theta \delta \omega }=\left[\hat{\omega }\times \right]\frac{\left[1-\cos(|\hat{\omega }|\Delta t)\right]}{|\hat{\omega }{|}^{2}}+I_{3}\Delta t-{\left[\hat{\omega }\times \right]}^{2}\frac{\left[|\hat{\omega }\left|\Delta t-\sin(\right|\hat{\omega }\left|\Delta t\right)\right]}{|\hat{\omega }{|}^{3}},\\ \; & \Phi_{\delta \omega \delta \theta }=0_{3},\\ \; & \Phi_{\delta \omega \delta \omega }=I_{3}. \end{array}$$

The state update is still performed using the approach in Equation (6). The angular velocity is assumed to be constant over the time-step.

The measurement update uses a similar form to the state-based approach, expressed as,

(35) $$\hat{b}=h\left(\Delta x\right)+v.$$

However, instead of considering the measured pixel coordinates, it considers the measured star unit vectors directly. The unit vectors are calculated using the image measurements α and γ using Equation (10). The measurement function is defined as,

(36) $$h\left(x\right)=\left\{\begin{array}{c} A\left(q\right)r_{1}\\ \vdots \\ A\left(q\right)r_{N} \end{array}\right\}.$$

The linearised measurement matrix is solved by considering the partial derivatives of the measurement function with respect to the state error vector,

(37) $$H=\frac{\partial h(x)}{\partial \Delta x}.$$

This is solved directly in [15], using the chain rule and so the state error vector is expressed as,

(38) $$H=\left[\begin{array}{cc} \left[A\left(q\right)r_{1}\times \right] & 0_{3}\\ \vdots & \vdots \\ \left[A(q\right)r_{N}\times ] & 0_{3} \end{array}\right].$$

The attitude error represents the difference between the true and estimate quaternion by the relation, assuming the attitude error is suitably small,

(39) $$q^{\mathrm{true}}=\hat{q}+\frac{1}{2}\Xi \left(\hat{q}\right)\delta \theta ,$$

where Ξ was defined in Equation (9). Similarly, the angular velocity is related by,

(40) $$\omega^{\mathrm{true}}=\hat{\omega }+\delta \omega .$$

The methodology of this approach is summarised in Table 2.

## 3. Results and Discussion

This section presents the results of analysing each estimation model. The results include simulations of the star tracker, as well as real night sky testing using the CROSS star tracker [30]. In the real night sky test, the Earth’s rotation is used for dynamic testing under a constant rotation. This unfortunately constrains performance analysis to precision only and not accuracy, but still allows meaningful conclusions to be derived.

### 3.1. Simulations

Both models presented in Section 2.3 are simulated and compared. The error models described in Section 2.2 are used to produce representative measurement errors. The model errors are the same for each filter approach considered. As mentioned, results are also compared to a static least square estimator, to establish a baseline for performance.

Each model starts at an initial attitude of $q_{0}=\frac{\sqrt{2}}{2}{\left\{\begin{array}{cccc} 0 & 0 & 1 & 1 \end{array}\right\}}^{T}$. The star tracker is set initially at a constant rotation in the *y*-axis, or pitch direction, with a speed of 36 arc-sec/s. A noise of $1\times 10^{-4}$ pixels is set at the centroid of each star.

The simulations are run over a period of 90 min, with measurements captured once every second, i.e., 1 Hz. The star tracker is set to a square field of view of 20${}^{\circ }$ and a stellar magnitude maximum of 5.0. The attitude does not considerably improve with more than 8–10 stars [15,31], so a higher magnitude limit is not necessary.

**Figure 2:**
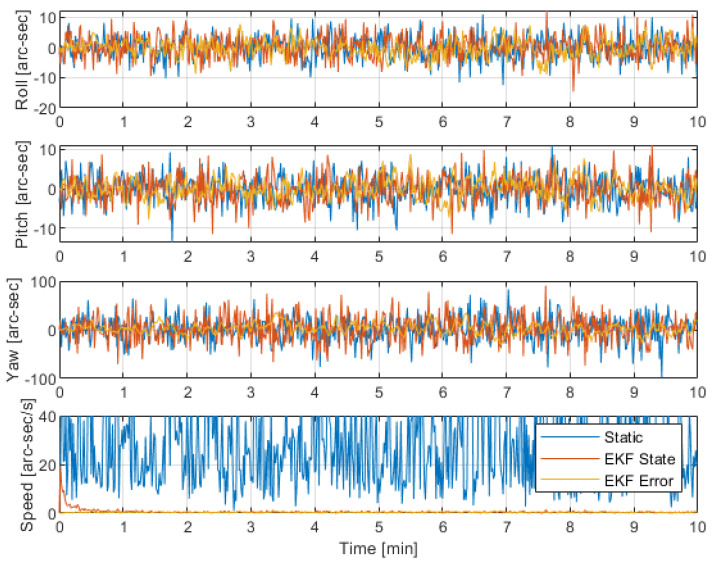
Attitude measurement performance for the simulation operating at a constant speed of 36 arc-sec/s.

The results are presented in Figure 2 and Table 3. The best performance for angular velocity is found for the state-based EKF compared to static LLS and state error. A slight improvement from state-based and a large improvement from static when comparing the speed error and standard deviation of Table 3. However, in terms of the attitude, the results are not so distinct.

The yaw error is clearly the worst, being the direction perpendicular to the boresight. In all directions, a slight improvement might be noted in the yaw error by adopting a state approach. The state error approach corrects by small increments at each time step. With significant noise in each stellar measurement, the restricted error term may not be enough to correct the noise.

### 3.2. Real Night Sky

The EKF approaches may also be compared and further analysed through samples of real night sky images. The EKF is also compared to a LLS based approach that does not consider any prior information.

**Figure 3:**
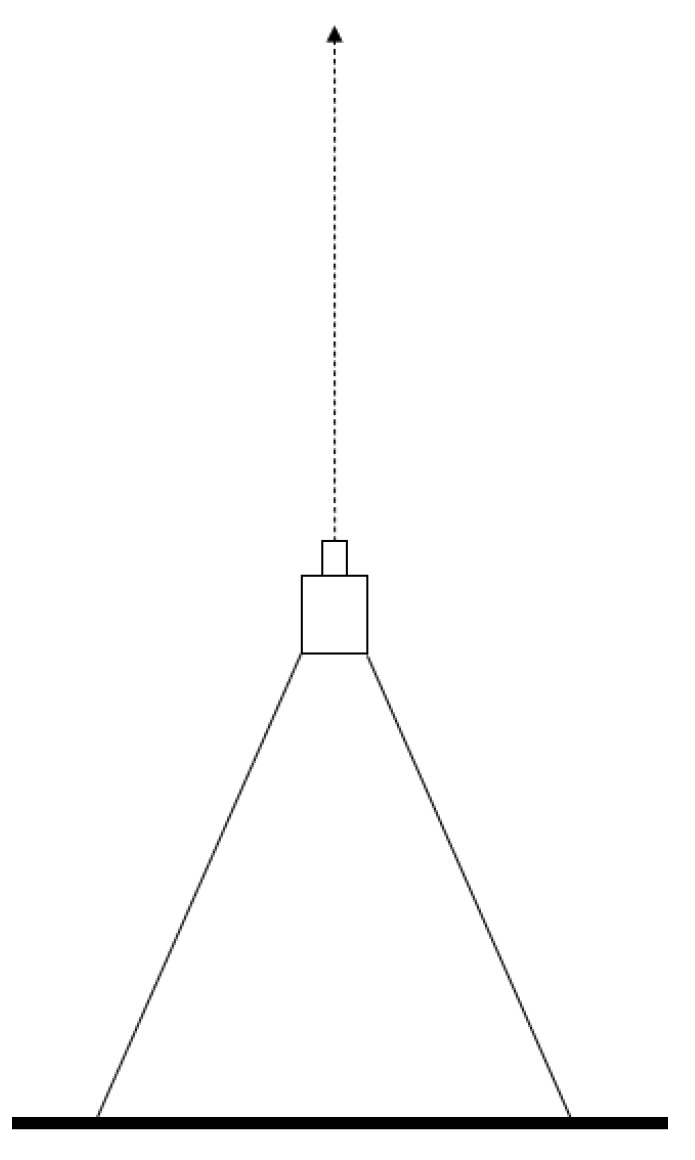
Illustration of zenith facing camera for static image testing using Earth rotation. Angular movement of stars through camera field of view caused solely by Earth rotation.

The test is performed by fixing the CROSS star tracker to a tripod and placing it with the camera facing approximately at the zenith. The camera’s horizontal field of view is approximately $19.9^{\circ }$ and the lens focus is located a distance to the sensor that intentionally blurs the stars in the image, aiding sub-pixel centroid analysis. After each captured image, each star is processed by measuring the unit pointing vector and identifying it to an equivalent catalogue of vectors. These vectors are then employed in the LLS or EKF. An illustration of the assembly is presented in Figure 3. As the Earth rotates, the stars move across the camera’s field of view at the known speed of Earth’s rotation.

To compute a constant attitude throughout the test duration, the Earth’s rotation, as well as empirically known nutation and precession angular fluctuations, may be corrected through a series of matrix operations to the measured attitude matrix. The residuals and precision of the measured attitude may then be calculated, providing an evaluation of the star tracker’s performance. The corrections to the attitude matrix *A* may be given by,

(41) $$A_{corr}=RNPA_{uncorr},$$

where *R*, *N* and *P* are the Earth rotation, nutation and precession matrices, respectively. Further description of each correction is described in [33]. The spread is calculated by fitting a linear trend line to the data set, measuring the goodness-of-fit, and transforming this to an error variance.

Each image was captured on a hill in the Inner West of the City of Sydney, Australia, on May 10, 2021. The sky contained some light pollution, but the astronomy weather forecast website, Meteo Blue, reported the presence of no planetary bodies in the night sky, with good visibility conditions and little to no cloud cover. It is considered that atmospheric noise may lead to worse performance, but should not significantly affect comparisons of each approach. The camera board is a FLIR Blackfly S containing a Sony IMX264 image sensor [35], 2448 × 2048 px, and is accompanied by a Scorpion Imaging lens [36], with an approximate diameter of 17 mm and 2/3″ sensor size. Each image is captured with a 15 s interval and an exposure time of 0.15 s.

Each approach is compared, also considering a shorter time difference between each image, and so a faster rotation speed. This is accounted for by reducing the time factor between each correction. Tthe measured attitude in each direction, yaw, pitch and roll, and the angular velocity is considered. The angular velocity of the LLS approach is calculated by taking the simple difference between the measured Euler angles and dividing by the time step.

It should be noted that the dependence on update rate has minimal influence on performance. Farrenkopf [15,37] calculated the steady-state error in the state error EKF for a gyroscope and star tracker Kalman filter, where,

(42) $$\sigma_{\theta }⟶\Delta t^{1/4}{\left(\sigma_{n}\sigma_{v}\right)}^{1/2},$$

where $\sigma_{v}$ is the gyroscope noise. For the gyroless case, $\sigma_{v}$ may be treated as a process noise in the propagation step. The dependence on the time update Δt is the same though, where it is the quartic root.

**Figure 4:**
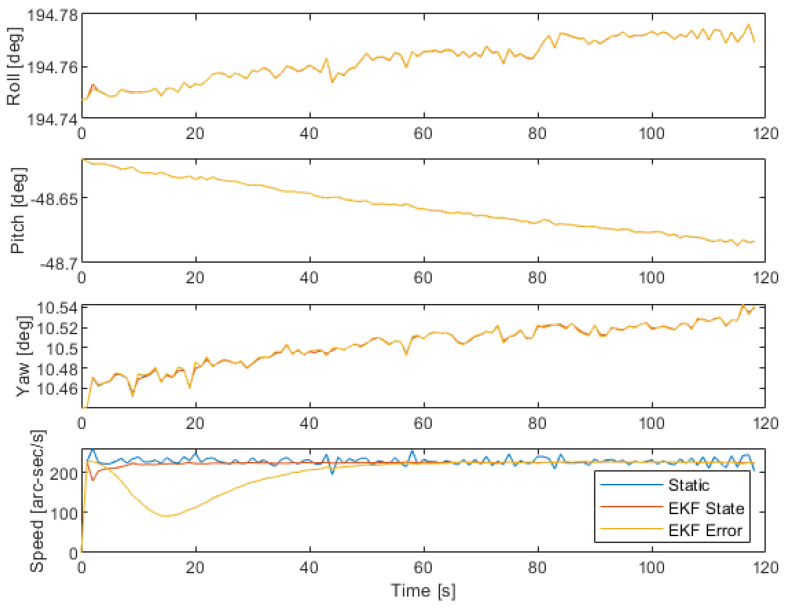
Attitude measurement performance for the standard time step of 1 s, considering each approach.

The results for the attitude and angular velocity are presented in Figure 4. The results are also tabulated in Table 4. The error is calculated for the attitude by fitting a trend line and considering the residual. The error in the angular velocity measurement is calculated by the residual to the known Earth rotation. The standard deviation represents the precision of the data set series to the trend line.

The attitude in Figure 4 shows a slow drift from the initial attitude. This may be due to external factors such as the wind or a general slip on the tripod, and is highlighted by the data spread being greater than the slip or drift speed. The speed results are calculated after a convergence time of 60 s.

Comparing the attitude results of the EKF to the LLS static approach, no distinct improvement is derived from using a filter. Differences between the state error and state-based approaches are also not so clear. This conclusion was also demonstrated in Section 3.1.

The convergence time of the angular velocity is much longer for the EKF, but especially for the state-error approach. This is reasonable, as the state error correction is restricted to only small increments. However, once converged, as seen in Table 4, the EKF precisions are much higher than the static LLS. It may be concluded that for high spin rates, a Kalman filter is more suitable to estimate angular velocity.

Overall, the state approach is better in terms of angular velocity, as in Table 4. This result is suspected to be due to the restricted magnitude of the state error, which is insufficient to correct for propagation approximation error. The difference in attitude accuracy and precision between state-based and state error techniques after convergence in Figure 4 and Table 4 is not strong enough to significantly prefer a state-based over a state error-based approach.

It could be argued that the star tracker could also be tested against more erratic behaviour with a changing angular velocity. However, such phenomena are almost always caused by a deliberate torque in the spacecraft attitude controller, which can be directly measured and fed into the attitude determination system. This would improve results. Higher speeds also are not appropriate, as significantly smeared images at high spin rates cannot successfully identify an attitude [38].

## 4. Conclusions

This work compares state and state error-based Kalman filter formulations to determine the best approach for the gyroless star tracker. Each approach is traditionally known as additive and multiplicative EKF, but is relabelled for the gyroless case where the spin rate is a directly estimated state. This treatment is important for new applications of star trackers in SSA, where spacecraft spin speeds are an important parameter, as well as traditional attitude determination.

Approaches consider both simulation and real night sky analysis. The real night sky case uses a novel technique for testing in the absence of a known truth orientation. The state-based Kalman approach was determined to perform the best in each case. It is not restricted by the error term and reset step. This approach may encounter ill conditioning of the covariance matrix and requires renormalisation, increasing the error margin. However, the magnitude of these errors are considered minor and were not noticeable in the results reported. Moreover, resetting the covariance matrix proved unnecessary for the analyses reported here.

Further work will see implementation of this Kalman filter on an upcoming satellite mission. It will also see implementation of the state estimate in direct SSA applications.

## Figures and Tables

**Table 1:** EKF methodology for the Star Tracker only state-based approach.

States	$x_{k}={\left\{\begin{array}{ccccccc} q_{0} & q_{1} & q_{2} & q_{3} & \omega_{x} & \omega_{y} & \omega_{z} \end{array}\right\}}^{T}$
	$z_{k}={\left\{\begin{array}{ccc} \alpha_{1} & \ldots & \gamma_{n} \end{array}\right\}}^{T}$
Propagation Update	${\hat{q}}_{k+1}^{-}=\Phi_{qq}q_{k}^{+}$
	${\hat{\omega }}_{k+1}^{-}={\hat{\omega }}_{k}^{+}$
	$P_{k+1}^{-}=\Phi P_{k}^{+}\Phi^{T}+Q$
	$\Phi =\left[\begin{array}{cc} \Phi_{qq} & \Phi_{q\omega }\\ 0 & I_{3} \end{array}\right]$
Measurement Update	$K_{k}=P_{k}^{-}H_{k}^{T}({\hat{x}}_{k}^{-}{\left[H_{k}\left({\hat{x}}_{k}^{-}\right)P_{k}^{-}H_{k}^{T}\left({\hat{x}}_{k}^{-}\right)+R_{k}\right]}^{-1}$
	${\hat{x}}_{k}^{+}={\hat{x}}_{k}^{-}+K_{k}\left[{\hat{z}}_{k}-h_{k}\left({\hat{x}}_{k}^{-}\right)\right]$
	$P_{k}^{+}=\left[I_{7}-K_{k}H_{k}\left({\hat{x}}_{k}^{-}\right)\right]P_{k}^{-}$
	$H_{k}\left({\hat{x}}_{k}^{-}\right)=\left[\begin{array}{ccccccc} \frac{\partial \alpha_{1}}{\partial q_{0}} & \frac{\partial \alpha_{1}}{\partial q_{1}} & \frac{\partial \alpha_{1}}{\partial q_{2}} & \frac{\partial \alpha_{1}}{\partial q_{3}} & \frac{\partial \alpha_{1}}{\partial \omega_{x}} & \frac{\partial \alpha_{1}}{\partial \omega_{y}} & \frac{\partial \alpha_{1}}{\partial \omega_{z}}\\ \vdots & \vdots & \vdots & \vdots & \vdots & \vdots & \vdots \\ \frac{\partial \gamma_{n}}{\partial q_{0}} & \frac{\partial \gamma_{n}}{\partial q_{1}} & \frac{\partial \gamma_{n}}{\partial q_{2}} & \frac{\partial \gamma_{n}}{\partial q_{3}} & \frac{\partial \gamma_{n}}{\partial \omega_{x}} & \frac{\partial \gamma_{n}}{\partial \omega_{y}} & \frac{\partial \gamma_{n}}{\partial \omega_{z}} \end{array}\right]$
	$h_{k}\left({\hat{x}}_{k}^{-}\right)=\left\{\begin{array}{c} \alpha_{1}\\ \vdots \\ \gamma_{n} \end{array}\right\}$
	${\hat{q}}_{k}^{+}=\frac{{\hat{q}}_{k}^{*+}}{|{\hat{q}}_{k}^{*+}|}$

**Table 2:** EKF methodology for the Star Tracker using the state error approach.

States	$x_{k}={\left\{\begin{array}{ccccccc} q_{0} & q_{1} & q_{2} & q_{3} & \omega_{x} & \omega_{y} & \omega_{z} \end{array}\right\}}^{T}$
	$\Delta x_{k}={\left\{\begin{array}{cccccc} \delta \theta_{x} & \delta \theta_{y} & \delta \theta_{z} & \delta \omega_{x} & \delta \omega_{y} & \delta \omega_{z} \end{array}\right\}}^{T}$
	$z_{k}={\left\{\begin{array}{ccc} b_{1}^{T} & \ldots & b_{n}^{T} \end{array}\right\}}^{T}$
Propagation Update	${\hat{q}}_{k+1}^{-}=\Phi_{qq}q_{k}^{+}$
	${\hat{\omega }}_{k+1}^{-}={\hat{\omega }}_{k}^{+}$
	$P_{k+1}^{-}=\Phi P_{k}^{+}\Phi^{T}+Q$
	$\Phi =\left[\begin{array}{cc} \Phi_{\delta \theta \delta \theta } & \Phi_{\delta \theta \delta \omega }\\ 0_{3} & I_{3} \end{array}\right]$
Measurement Update	$K_{k}=P_{k}^{-}H_{k}^{T}({\hat{x}}_{k}^{-}{\left[H_{k}\left({\hat{x}}_{k}^{-}\right)P_{k}^{-}H_{k}^{T}\left({\hat{x}}_{k}^{-}\right)+R_{k}\right]}^{-1}$
	$\Delta {\hat{x}}_{k}^{+}=K_{k}\left[{\hat{z}}_{k}-h_{k}\left({\hat{x}}_{k}^{-}\right)\right]$
	$P_{k}^{+}=\left[I_{7}-K_{k}H_{k}\left({\hat{x}}_{k}^{-}\right)\right]P_{k}^{-}$
	$H_{k}\left({\hat{x}}_{k}^{-}\right)=\left[\begin{array}{cc} \left[A\left({\hat{q}}_{k}^{-}\right)r_{1}\times \right] & 0_{3}\\ \vdots & \vdots \\ {\hat{q}}_{k}^{-})r_{N}\times ] & 0_{3} \end{array}\right]$
	$h_{k}\left({\hat{x}}_{k}^{-}\right)=\left\{\begin{array}{c} A\left({\hat{q}}_{k}^{-}\right)r_{1}\\ \vdots \\ A\left({\hat{q}}_{k}^{-}\right)r_{N} \end{array}\right\}$
	$q_{k}^{*+}={\hat{q}}_{k}^{-}+\frac{1}{2}\Xi \left({\hat{q}}_{k}^{-}\right)\delta \theta_{k}^{+}$
	${\hat{q}}_{k}^{+}=\frac{{\hat{q}}_{k}^{*+}}{|{\hat{q}}_{k}^{*+}|}$
	${\hat{\omega }}_{k}^{+}={\hat{\omega }}_{k}^{-}+\delta {\hat{\omega }}_{k}^{+}$

**Table 3:** EKF results with the simulation using an angular speed of 36 arc-sec/s.

Parameter	Measure	LLS	EKF State	EKF State Error
Roll	Error [arc-sec]	0.04	0.01	0.12
	Standard Deviation [arc-sec]	3.59	2.60	3.26
Pitch	Error [arc-sec]	0.02	0.05	0.13
	Standard Deviation [arc-sec]	3.65	2.53	2.91
Yaw	Error [arc-sec]	0.60	0.46	0.20
	Standard Deviation [arc-sec]	25.86	7.88	12.93
Speed	Error [arc-sec/s]	30.63	0.12	0.4
	Standard Deviation [arc-sec/s]	21.3	0.31	0.18

**Table 4:** EKF results with real night sky imagery using a 1 s time displacement between each image, and so an angular speed of 36 arc-sec/s.

Parameter	Measure	LLS	EKF State	EKF State Error
Roll	Residual [arc-sec]	28.49	28.32	28.51
	Standard Deviation [arc-sec]	8.34	8.43	8.34
Pitch	Residual [arc-sec]	66.28	66.46	66.30
	Standard Deviation [arc-sec]	6.26	6.33	6.27
Yaw	Residual [arc-sec]	75.39	75.89	75.06
	Standard Deviation [arc-sec]	27.87	26.18	27.74
Speed	Residual [arc-sec/s]	10.17	0.70	1.50
	Standard Deviation [arc-sec/s]	8.09	0.61	1.16

## References

[1] Kalman R.E. (1960). A new approach to linear filtering and prediction problems. J. Fluids Eng. Trans. ASME.

[2] McLean J.D., Schmidt S.F., McGee L.A. (1961). Optimal Filtering and Linear Prediction Applied to a Midcourse Navigation System for the Circumlunar Mission.

[3] Smith G.L., Schmidt S.F., McGee L.A. (1962). Application of Statistical Filter Theory to the Optimal Estimation of Position and Velocity on Board a Circumlunar Vehicle.

[4] Farrell J.L. (1970). Attitude Determination by Kalman Filtering. Autonatica.

[5] Pauling D.C., Jackson D.B., Brown C.D. SPARS algorithms and simulation results. Proceedings of the Symposium on Spacecraft Attitude Determination.

[6] Gai E., Daly K., Harrison J., Lemos L. (1985). Star-Sensor-Based Satellite Attitude/Attitude Rate Estimator. J. Guid. Control Dyn..

[7] Challa M., Natanson G.A., Baker D.E., Deutschmann J.K. Advantages of Estimating Rate Corrections During Dynamic Propagation of Spacecraft Rates-Applications to Real-Time Attitude Determination of SAMPEX. Proceedings of the Flight Mechanics/Estimation Theory Symposium.

[8] Chu D., Harvie E. Accuracy of the ERBS Definitive Attitude Determination System in the Presence of Propagation Noise. Proceedings of the Flight Mechanics/Estimation Theory Symposium.

[9] Crassidis J.L., Markley F.L. (1997). Predictive Filtering for Attitude Estimation without Rate Sensors. J. Guid. Control Dyn..

[10] Hajiyev C., Guler D.C. (2017). Review on gyroless attitude determination methods for small satellites. Prog. Aerosp. Sci..

[11] Shuster M.D. (1990). Kalman Filtering of Spacecraft Attitude and the QUEST Model. J. Astronaut. Sci..

[12] Leffens E.J., Markley F.L., Shuster M.D. (1982). Kalman filtering for spacecraft attitude estimation. J. Guid. Control Dyn..

[13] Crassidis J.L., Markley F.L. (2003). Unscented filtering for spacecraft attitude estimation. J. Guid. Control Dyn..

[14] Cheng Y., Crassidis J.L., Markley F.L. (2006). Attitude Estimation for Large Field-of-View Sensors. J. Astronaut. Sci..

[15] Markley F.L., Crassidis J.L. (2014). Filtering for Attitude Estimation and Calibration. Fundamentals of Spacecraft Attitude Determination and Control.

[16] Darling J.E., Houtz N., Frueh C., Demars K.J. Recursive filtering of star tracker data. Proceedings of the AIAA/AAS Astrodynamics Specialist Conference.

[17] Li J., Wei X., Zhang G. (2017). An extended Kalman filter-based attitude tracking algorithm for star sensors. Sensors.

[18] Tan N.D., Vinh T.Q., Tuyen B.T. (2016). A new approach for small satellite gyroscope and star tracker fusion. Indian J. Sci. Technol..

[19] Fan C., Meng Z., Liu X. Multiplicative quaternion extended consensus Kalman filter for attitude and augmented state estimation. Proceedings of the Chinese Control Conference, CCC.

[20] Lee D., Vukovich G., Lee R. (2017). Robust Adaptive Unscented Kalman Filter for Spacecraft Attitude Estimation Using Quaternion Measurements. J. Aerosp. Eng..

[21] Grewal M., Shiva M. Application of Kalman filtering to gyroless attitude determination and control system for environmental satellites. Proceedings of the 1995 34th IEEE Conference on Decision and Control.

[22] Dave S., Clark R., Lee R.S. (2022). RSOnet: An Image-Processing Framework for a Dual-Purpose Star Tracker as an Opportunistic Space Surveillance Sensor. Sensors.

[23] Tetlow M.R., Chin T. Robust Attitude Estimation to Support Space Monitoring Using Nano-Satellites. Proceedings of the AIAA SPACE 2014 Conference and Exposition.

[24] Gaoxiang O., Wenliang L., Pingke D., Guocan Z. Attitude and Angle Rate Determination of Gyroless Spacecraft Based on SVD Kalman Filter Only Using Star Sensor. Proceedings of the 2021 33rd Chinese Control and Decision Conference (CCDC).

[25] Leeghim H., Bang H., Lee C.Y. (2018). Angular rate and alignment estimation for gyroless spacecraft by only star trackers. Int. J. Control Autom. Syst..

[26] Ding Y., Zhao X., Zhang Z., Cai W., Yang N., Zhan Y. (2021). Semi-supervised locality preserving dense graph neural network with ARMA filters and context-aware learning for hyperspectral image classification. IEEE Trans. Geosci. Remote. Sens..

[27] Carron A., Todescato M., Carli R., Schenato L., Pillonetto G. Machine learning meets Kalman filtering. Proceedings of the 2016 IEEE 55th Conference on Decision and Control (CDC).

[28] Zhang S.T., Wei X.Y. Fuzzy adaptive Kalman filtering for DR/GPS. Proceedings of the 2003 International Conference on Machine Learning and Cybernetics (IEEE Cat. No. 03EX693).

[29] Russo A., Lax G. (2022). Using Artificial Intelligence for Space Challenges: A Survey. Appl. Sci..

[30] Suntup M., Cairns I., Critchley-Marrows J., Wu X., Albertson D., Guinane J., Jarvis B. Implementation of a WFOV Star Tracker in CubeSat and Small Satellite Attitude Determination Systems. Proceedings of the 43rd COSPAR Scientific Assembly.

[31] Critchley-Marrows J., Wu X. Investigation into Integrated Attitude Determination in High-Precision CubeSats. Proceedings of the 68th International Astronautical Congress.

[32] Critchley-Marrows J., Wu X. (2019). Investigation into Star Tracker Algorithms using Smartphones with Application to High-Precision Pointing CubeSats. Trans. Jpn. Soc. Aeronaut. Space Sci..

[33] Jarvis B., Guinane J., Wu X., Critchley-Marrows J. Development of a Low-Cost Testing Methodology for Star Trackers. Proceedings of the 19th Australian Space Research Conference.

[34] Chou J.C. (1992). Quaternion Kinematic and Dynamic Differential Equations. IEEE Trans. Robot. Autom..

[35] FLIR Technical Reference—FLIR Blackfly S. https://www.eureca.de/files/pdf/optoelectronics/flir/BFS-U3-50S5-BD2-Technical-Reference.pdf.

[36] Scorpion Vision Limited Specification Sheet—S-Mount 25mm F1.7 Lens–SVL-IR2517B5M. https://files.ecommercedns.uk/230067/ffc50f57ca9f2b26564af2e7b3343768.pdf.

[37] Farenkopf R.L. (1978). Analytic steady-state accuracy solutions for two common spacecraft attitude estimators. J. Guid. Navig. Control.

[38] Guinane J., Jarvis B., Suntup M., Wu X., Critchley-Marrows J., Cairns I.H. Assessing the viability of a wide field of view based stellar gyroscope. Proceedings of the 18th Australian Space Research Conference.

